# An Observational Study of Reduction in Glycemic Parameters and Liver Stiffness by Saroglitazar 4 mg in Patients With Type 2 Diabetes Mellitus and Nonalcoholic Fatty Liver Disease

**DOI:** 10.7759/cureus.9065

**Published:** 2020-07-08

**Authors:** Asis Mitra

**Affiliations:** 1 Internal Medicine, Ruby General Hospital, Kolkata, IND

**Keywords:** saroglitazar, dyslipidemia, nonalcoholic fatty liver disease (nafld), peroxisome proliferator-activated receptor (ppar), type 2 diabetes mellitus (t2dm)

## Abstract

Background

Metabolic abnormal conditions, such as diabetes and high triglycerides (TGs), are commonly associated with nonalcoholic fatty liver disease (NAFLD). Currently, there is no approved pharmacotherapy for NAFLD. Saroglitazar, the world’s first approved dual peroxisome proliferator-activated receptors (PPAR) α and γ agonist, was approved in India for the treatment of diabetic dyslipidemia. The objective of this study was to observe the safety and effectiveness of saroglitazar, 4 mg once daily, in reducing glycemic parameters and liver fibrosis in type 2 diabetes mellitus (T2DM) patients with NAFLD.

Method

In this prospective observational study, we enrolled 30 patients with T2DM and NAFLD (primarily detected by ultrasonography (USG) of the abdomen) who were treated with saroglitazar, 4 mg once daily, and the follow-up data were available for six months after saroglitazar treatment. During follow up, all patients were on stable antidiabetic and statin therapy. Liver stiffness was measured by FibroScan® (Echosens™ North America, Waltham, MA) elastography at baseline and at the six-month follow-up.

Results

At the six-month follow-up after saroglitazar treatment, significant improvement was observed in glycemic parameters, liver stiffness on FibroScan, and serum transaminase levels. The serum TG levels were significantly reduced with saroglitazar. No major adverse event was reported.

Conclusion

In this observational study of patients with T2DM and NAFLD, saroglitazar improved liver stiffness, as well as the glycemic and lipid parameters. A long-term randomized controlled clinical trial is required to further establish the safety and efficacy of saroglitazar in the treatment of T2DM and NAFLD.

## Introduction

Dyslipidemia and type 2 diabetes mellitus (T2DM) are the most commonly associated comorbid conditions seen in patients with metabolic disorders and cardiovascular diseases (CVD). As per the International Diabetes Federation (IDF), India has almost 77 million diabetic patients and is further set to increase to almost 134.2 million by the year 2045 [1]. Diabetes is associated with multiple comorbidities, such as chronic hyperglycemia, dyslipidemia, and hypertension. It is also associated with a non-cardiovascular disease like nonalcoholic fatty liver disease (NAFLD). NAFLD is a condition wherein symptoms do not appear at an early stage. In the late-stage, it can cause cirrhosis of the liver which leads to liver transplantation [2]. Insulin resistance has been considered as a core pathophysiological factor in T2DM and NAFLD [1, 3]. Insulin resistance is a major connecting factor between NAFLD and T2DM, and one study has shown that more than seven out of 10 patients of T2DM will have a comorbidity of NAFLD [4]. Early detection of NAFLD may be useful but challenging to identify those with potentially silent progressive fatty liver disease. Currently, liver biopsy remains the gold standard for NAFLD diagnosis, but it is an impractical and expensive tool to be considered as a routine test [5]. Routine liver enzymes and advanced FibroScan® (Echosens™ North America, Waltham, MA) test methods can help to evaluate progress in NAFLD management [4, 6]. Currently, there is no Food and Drug Administration (FDA) approved drug available for the treatment of NAFLD. Saroglitazar is the first dual peroxisome proliferator-activated receptor (PPAR) α/γ agonist. It was approved in India for the treatment of diabetic dyslipidemia in 2013 and has shown a reducing effect on elevated liver enzymes in T2DM patients [7]. Herein, we have prospectively analyzed the data of 30 diabetic patients with NAFLD who were treated with saroglitazar, 4 mg once daily, for a period of six months.

## Materials and methods

After obtaining institutional ethical committee clearance (RGH/KOL/EC/nonspon/06/11-2018) from Ruby General Hospital, we prospectively enrolled 30 T2DM patients, aged 18 years and above, who were on treatment with either injectable or oral anti-diabetic agents at the time of enrollment. The subjects were enrolled from December 2018 to November 2019. The primary inclusion criteria were patients having T2DM and NAFLD documented by ultrasonography (USG) of the abdomen. Exclusion criteria were type 1 diabetes mellitus, pregnancy, established chronic liver disease (including hepatitis B and C), chronic kidney disease as evidenced by serum creatinine more than 1.5 mg/dL, and patients with alcoholic liver disease.

Once abdominal USG screening was done, all patients underwent evaluation for fibrosis grading and liver stiffness measured by the FibroScan machine. Essentially, this technology measures the velocity of the sound wave passing through the liver and then converts that measurement into a liver stiffness measurement (measured as kilopascal (kPa)); the entire process is often referred to as liver ultrasonographic elastography. Once the patient’s NAFLD condition was confirmed, then saroglitazar magnesium, 4 mg once daily (OD), was prescribed for six months. In this study, saroglitazar was prescribed to these 30 subjects at the discretion of the treating physician, as per prescribing information, without doing any specific experimentations. All patients were followed up after six months, primarily to measure changes in blood sugar, lipid profile, and liver fibrosis score using the FibroScan. The ongoing medications remained unchanged throughout the study period of six months. Statistical analyses were done using GraphPad InStat® (GraphPad Software, San Diego, CA), and results were analyzed applying paired t-test. A p-value of < 0.05 was considered statistically significant.

## Results

A total of 30 T2DM patients (male:female ratio 7:3) having NAFLD were enrolled in this study. At baseline, the mean age of patients was 51.5 years with the average duration of diabetes of 9.6 years (Table 1). All patients were already receiving a stable dose of anti-diabetic medications. During the follow-up period of six months, all patients were advised to continue with a similar dose of antidiabetic drugs, statins, along with saroglitazar, 4 mg OD. All subjects were evaluated prospectively from baseline to six months with the treatment of saroglitazar, 4 mg once daily. At the baseline, out of a total of 30 patients, 11 patients had fibrosis F3 grade (9.5 - 12.4 kPa) and 19 patients had a fibrosis F4 grade (≥ 12.5 kPa). At the six-month follow-up after saroglitazar therapy, the glycosylated hemoglobin (HbA1c) level was significantly reduced from 8.14 ± 0.52% to 7.74 ± 0.53% (P < 0.0001). Other blood glucose parameters, like fasting blood sugar (FBS) and postprandial blood sugar (PPBS), were also reduced significantly from baseline to the end of the six-month period of follow-up (Table 2). Amongst lipid parameters, serum triglyceride (TG) was significantly reduced from 179.4 ± 38.33 mg/dL to 112.33 ± 26.82 mg/dL (P < 0.0001) along with a significant reduction in total cholesterol (TC) and low-density lipoprotein (LDL) at six-month follow-up (Table 2). The liver enzymes, alanine aminotransferase (ALT), aspartate aminotransferase (AST), gamma-glutamyl transferase (GGT), and alkaline phosphatase (ALP), were also reduced significantly from baseline at the six-month follow-up after saroglitazar treatment (Table 2). On the FibroScan evaluation, liver stiffness was significantly reduced from 13.933 ± 2.87 kPa at baseline to 8.503 ± 1.86 kPa at the six-month follow-up (with a mean difference of -5.43; p-value < 0.0001). At the six-month follow-up, seven patients had fibrosis grade F1 (5.6 - 7.1 kPa), 13 patients had fibrosis grade F2 (7.2-9.4 kPa), eight patients had fibrosis F3 grade, and two patients had fibrosis F4 grade (Table 3). 

**Table 1:** Patients Baseline Characteristics ARBs: angiotensin receptor blockers; CCBs: calcium channel blockers; CKD: chronic kidney disease; DM: diabetes mellitus; DPP4: dipeptidyl peptidase 4; eGFR: epidermal growth factor receptor; F: female; M: male; SD: standard deviation; SGLT2: sodium-glucose cotransporter-2

No. of patients (n)	30
Age (years); Mean ± SD	51.5 ± 9.1
Gender ratio (M: F)	7:3
Average duration of DM (years)	9.6
Other comorbid conditions	n (%)
Hypertension	20 (67)
Dyslipidemia	18 (60)
Elevated triglyceride	7 (27)
CKD (eGFR < 60 mL/min/m^2^)	8 (27)
Hypothyroidism	1 (3)
Gout	1 (3)
Ongoing medications	n (%)
Metformin	12 (40)
Sulfonylureas	12 (40)
DPP4 inhibitors	11 (37)
SGLT2 inhibitors	5 (16)
Insulin	10 (33)
Atorvastatin	3 (10)
Rosuvastatin	11 (37)
ARBs	9 (30)
CCBs	4 (13)
Others	3 (10)

**Table 2:** Change in Biochemical/Clinical Parameters and Liver Stiffness Values at Six-Month Follow-up After Treatment With Saroglitazar, 4 mg Once Daily ALT: alanine aminotransferase; AST: aspartate aminotransferase; EMI: body mass index; eGFR: epidermal growth factor receptor; FBS: fasting blood sugar; GGT: gamma-glutamyl transferase; HbA1c: glycosylated hemoglobin; HDL: high-density lipoprotein; kPa: kilopascal; LDL: low-density lipoprotein; PPBS: postprandial blood sugar; TC: total cholesterol; TG: triglycerides

Laboratory Parameters	Baseline	6 months follow-up	Mean Difference	95% CI	P value
HbA1c (%)	8.14 ± 0.52	7.74 ± 0.53	-0.397	(-0.489 to -0.304)	< 0.0001
FBS (mg/dL)	146.83 ± 23.3	124.17 ± 14.1	-22.67	(-30.79 to -14.55)	< 0.0001
PPBS (mg/dL)	245.23 ± 32.71	213.73 ± 30.67	-31.5	(-39.33 to -23.67)	< 0.0001
TC (mg/dL)	188.27 ± 22.90	177.16 ± 19.51	-11.10	(-13.13 to -9.07)	< 0.0001
LDL (mg/dL)	122.43 ± 18.85	112.9 ± 15.9	-9.53	(-11.05 to -7.95)	< 0.0001
HDL (mg/dL)	39.75 ± 7.2	39.77 ± 6.6	0.02	(-0.97 to 0.97)	1.0
TG (mg/dL)	179.4 ± 38.33	112.33 ± 26.82	-67.07	(-78.45 to -55.68)	< 0.0001
ALT (mg/dL)	56.47 ± 15.17	42.3 ± 11.26	-14.17	(-17.87 to -10.46)	< 0.0001
AST (mg/dL)	48.57 ± 13.15	36.63 ± 8.14	-11.93	(-15.25 to -8.62)	< 0.0001
GGT (mg/dL)	54.97 ± 9.52	45.33 ± 5.94	-9.63	(-12.32 to -6.95)	< 0.0001
Alkaline Phosphatase (mg/dL)	110.93 ± 19.99	91.83 ± 15.39	-19.1	(-24.07 to -14.13)	< 0.0001
Liver stiffness (kPa)	13.93 ± 2.87	8.50 ± 1.86	-5.43	(-5.94 to -4.92)	< 0.0001
eGFR (ml/min/kg^2^)	80.38 ± 19.58	81.07 ± 19.29	0.68	(0.25 to 1.11)	0.003
BMI (kg/m^2^)	28.607	28.307	-0.3	(-0.38 to -0.22)	< 0.0001

**Table 3:** Number of Patients With Fibrosis F0 to F4 Grade at Baseline and Six-Month Follow-up kPa: kilopascal

Fibrosis Grade	Baseline	Six-month follow-up
F0 (< 5.5 kPa)	0	0
F1 (5.6 - 7.1 kPa)	0	7
F2 (7.2 - 9.4 kPa)	0	13
F3 (9.5 - 12.4 kPa)	11	8
F4 (≥ 12.5 kPa)	19	2

The mean estimated glomerular filtration rate (eGFR) level was improved by 0.68 ml/min/kg^2^ and the mean body mass index (BMI) was reduced by 0.3 kg/m^2^ during the study (Table 2). No any major or serious adverse events were reported during the six-month follow-up period.

## Discussion

Insulin resistance is a pathological root cause factor for T2DM and is commonly associated with metabolic conditions, such as dyslipidemia and elevated triglycerides (TG). Uncontrolled TG accumulates inside hepatic tissue and leads to steatosis changes which are considered as nonalcoholic fatty liver disease (NAFLD) [8]. Currently, in India, ~77 million people are suffering from T2DM, and data from India also indicate that around 90% of Indian diabetics also suffer from associated lipid abnormalities [1]. The dyslipidemia pattern in Indian adults is different, wherein the most common type is low HDL, followed by high TG, high cholesterol, and high LDL [9].

Many observational studies have shown that high TG is a cardiovascular disease (CVD) risk factor [10-11]. In 2015, a study analyzed the results of two trials, the Study of RO4607381 in Stable Coronary Heart Disease Patients With Recent Acute Coronary Syndrome (dal-OUTCOMES) and Myocardial Ischemia Reduction with Aggressive Cholesterol Lowering (MIRACL), which showed that high TG levels (> 175 mg/dL on the long-term and > 195 mg/dL on short-term, respectively) in spite of statin therapy in post-acute coronary syndrome (ACS) patients led to an increased CVD risk (60% and 50% higher on long- and short-term, respectively) compared to those patients who had lower TG levels (≤ 80 mg/dL on long-term and ≤ 135 mg/dL on short-term) [12]. In a recently published study, increased residual CV risk in patients with diabetes and high TG levels (200 - 499 mg/dL), despite statin-controlled LDL cholesterol (< 100 mg/dL), was observed compared to normal TG levels (< 150 mg/dL) [13].

The Reduction of Cardiovascular Events with Icosapent Ethyl Intervention Trial (REDUCE-IT) study has concluded that among patients with elevated triglyceride levels despite the use of statins, the risk of ischemic events (including cardiovascular death) was significantly lower among those who received 2 g of icosapent ethyl twice daily, which is considered a TG lowering agent [14]. The European Society of Cardiology (ESC) 2019 recommended that in high-risk patients with TG levels between 135 - 499 mg/dL, despite statin treatment, TG lowering drugs should be considered in combination with a statin [15].

Saroglitazar is a novel dual PPAR α/γ agonist with a predominant PPAR α agonistic activity that was approved in 2013 by the Drug Controller General of India (DCGI) for management of diabetic dyslipidemia and hypertriglyceridemia in T2DM patients not controlled by statins alone. Being a dual PPAR agonist, in many clinical studies, a significant reduction of triglycerides (due to PPAR α agonist) and HbA1c reduction (due to PPAR γ agonist) has been shown[16-17]. In a recently published study by Kaul et al., saroglitazar showed a reduction in TG in the range from 45% - 62%, HbA1c from 0.7% - 1.6%, and small dense low-density lipoprotein (sd-LDL) by 20.3% in ~5,800 diabetic dyslipidemia patients [7]. Recently, Jain et al. published a first-ever human euglycemic clamp study of saroglitazar in T2DM patients with elevated triglyceride levels [18]. At the end of four months, it was observed that saroglitazar had reduced TG, HbA1c, and fasting plasma glucose (FPG) significantly from baseline. In addition, it also improved the insulin sensitivity index from 2.9 to 6.1 (M/I) (p < 0.05) and glucose metabolism from 1.6 to 3.2 (M) (p < 0.05) [18]. In this 30 patient observational study, saroglitazar showed similar results in mean TG reduction (-67 mg/dl) and HbA1c reduction (-0.4%) without showing any effect on kidney function or any other adverse events during six months of follow-up.

NAFLD is a condition that usually remains asymptomatic at initial stages and is characterized by the accumulation of fat in the liver due to metabolic conditions. It has been associated with risk factors, such as metabolic syndrome (MS), insulin resistance (IR), altered gut flora, and persistent inflammation. About 3% - 15% of cases of the obese, non-alcoholic steatohepatitis (NASH) patients progress to cirrhosis and about 4% - 27% of NASH with cirrhosis cases transform to hepatocellular carcinoma (HCC) [19]. Amongst various risk factors, obesity is most commonly diagnosed with NAFLD by ~70% and NASH by ~33%. The second most common risk factor is T2DM with a 30% - 70% prevalence of NAFLD which is more prone to malignancies [20-21]. A meta-analysis conducted in Europe, United States, and Asia depicted a 7% increase in relative risk (RR) of developing HCC in overweight patients and further higher RR in obese patients by 85% [22]. Detection of NAFLD is a real challenge as, in the majority of cases, it remains asymptomatic. The European or American Association of the Study of Liver (EASL/AASLD) laid down the non-invasive criteria for a diagnosis like fibrosis 4 (FIB-4), NAFLD fibrosis score (NFS) [23]. At initial stages, the elevation of liver enzymes, like AST, ALT, GGT, and its ratio, indicates the need for further evaluation (like USG) and is not able to detect fibrosis. USG-based elastography techniques, such as transient elastography, acoustic radiation force impulse, and shear wave elastography, have been used for the estimation of fat and fibrosis with promising results. Biopsy is a gold standard method to differentiate between NASH and HCC [23]. Currently, there is no FDA-approved drug available for the treatment of NASH and NAFLD. Metformin, vitamin E, pioglitazone, and glucagon-like peptide-1 (GLP-1) agonists are commonly used as off-label medications. Saroglitazar magnesium in patients with nonalcoholic fatty liver disease and/or nonalcoholic steatohepatitis (EVIDENCES IV), a phase II clinical study conducted in the USA (NCT03061721), showed that saroglitazar, 4 mg, resulted in significant ALT reduction by 44% from baseline in NAFLD patients. The results also showed a significant reduction in liver fat content on magnetic resonance imaging-derived proton density fat fraction (MRI-PDFF) (Gawrieh S, Noureddin M, Loo NM, Mohseni R, Awasty VR, Cusi K, Kowdley KV, Lai M, Schiff ER, Parmar DV, Patel PR, Chalasani NP: Abstract LO10: A phase 2, prospective, multicenter, double-blind, randomized study of saroglitazar magnesium 1 mg, 2 mg or 4 mg versus placebo in patients with nonalcoholic fatty liver disease and/or nonalcoholic steatohepatitis (EVIDENCES IV). Presented at the American Assn. for the Study of Liver Diseases annual meeting, Nov. 8-12, 2019, Boston, MA).

In our observation, saroglitazar, 4 mg once daily, showed a significant reduction in ALT, AST, and GGT levels at the six-month follow-up compared to baseline. In this study, all patients underwent liver stiffness measurement on fibrosis and the results showed a significant reduction from 13.9 kPa at baseline to 8.5 kPa at the six-month follow-up (p < 0.0001). The drug was found to be safe, well-tolerated, and without any major adverse event reported during the six-month follow-up.

Limitations of this study

This was an observational study conducted at a single center with a small sample size.

## Conclusions

In this observational study, treatment with saroglitazar, 4 mg once daily for six months, resulted in significant improvement in liver biochemical parameters and liver stiffness measured by the FibroScan, along with significant improvement seen in glycemic and lipid parameters in patients with T2DM and NAFLD. Therefore, it is a very useful oral hypoglycemic agent that was studied in the Indian population and needs further study among T2DM patients throughout the world.
